# Behavioral responses to transfluthrin by *Aedes aegypti*, *Anopheles minimus*, *Anopheles harrisoni*, and *Anopheles dirus* (Diptera: Culicidae)

**DOI:** 10.1371/journal.pone.0237353

**Published:** 2020-08-12

**Authors:** Chutipong Sukkanon, Jirod Nararak, Michael John Bangs, Jeffrey Hii, Theeraphap Chareonviriyaphap

**Affiliations:** 1 Department of Entomology, Faculty of Agriculture, Kasetsart University, Bangkok, Thailand; 2 HydroSciences Montpellier (HSM), Institut de Recherche pour le Développement (IRD), CNRS, Université Montpellier, Montpellier, France; 3 Public Health & Malaria Control Department, PT Freeport Indonesia/International SOS, Kuala Kencana, Papua, Indonesia; 4 Malaria Consortium Asia Regional Office, Faculty of Tropical Medicine, Mahidol University Bangkok, Bangkok, Thailand; 5 College of Public Health, Medical & Veterinary Sciences, James Cook University, North Queensland, Australia; National Taiwan Ocean University, TAIWAN

## Abstract

Airborne spatial repellency (SR) is characterized and distinguished from other chemical actions including contact locomotor excitation and toxicity. The use of volatile spatial repellents is a potential new intervention class for combatting mosquito-borne pathogen transmission; therefore, continuing investigations on the actions of these chemicals that modify mosquito host‐seeking behavior (i.e., bite prevention) is needed. The objective of this study is to characterize the key behavioral avoidance actions of transfluthrin (TFT) to advance spatial repellent development into practical products. Behavioral avoidance responses were observed for adult laboratory strains of *Aedes aegypti*, *Anopheles minimus* and *An*. *dirus*, and two field populations of *An*. *harrisoni* and *Ae*. *aegypti*, respectively. Established TFT sublethal (LC_50_ and LC_75_), lethal concentrations (LC_99_) and discriminating concentrations (DCs) were selected corresponding to each mosquito test species. Spatial repellency and contact excitation (‘irritancy’) responses on adult mosquitoes to TFT were assessed using an excito-repellency assay system. At LC_50_, TFT exhibited strong avoidance with *An*. *minimus* (60.1% escape) and *An*. *dirus* (80% escape) laboratory strains, showing between 12 and 16x greater escape response than *Ae*. *aegypti* (5% escape). Repellency responses for field collected *Ae*. *aegypti* and *An*. *harrisoni* were 54.9 and 47.1% escape, respectively. After adjusting the initial contact escape response (a measure of combined irritancy and repellency) to estimate only escape due to contact, the LC_50_ and LC_99_ showed moderate escape irritancy with laboratory *Ae*. *aegypti* (41.4% escape) and no contact activity against the field population. Adjustment showed only weak contact activity (16.1% escape) in laboratory *An*. *minimus* at LC_50_. Spatial repellency is the predominant mode of action of TFT among colonized and field mosquitoes used in this study. Established baseline (susceptible) dose-response curves assist in optimizing SR products for mosquito control and pathogen transmission prevention.

## Introduction

More than 4 billion people, primarily residing in tropical and sub-tropical countries, are at risk of infection from mosquito-borne diseases. For example, globally, an estimated 390 million dengue infections occur and around 200 million malaria cases are reported annually [1, 2]. Dengue and other *Aedes*-borne viruses (yellow fever, chikungunya, Zika) are projected to expand and pose a greater risk, both geographically and demographically, in the decades ahead [1]. While malaria programs have recently made substantial progress in reducing disease burden worldwide, the fight is far from over. In Thailand, 85,849 dengue infections were reported in 2018, while malaria contributed 6,625 cases, of which 82% were *Plasmodium vivax* infections, a latent relapse form of parasite complicating elimination goals in the country [3]. Even though malaria cases decreased 2.2-fold in 2018 from 2017, the actual number of cases are underreported [3].

Mosquito vector control through the use of insecticide-treated bed nets (ITNs) and indoor residual spraying (IRS) are considered to have made major contributions towards the reduction in the global malaria burden since 2000 [4]. Among the available AIs, synthetic pyrethroids remain the third most common class of chemicals for public health use worldwide during 2000–2009 [5], including Thailand [6], due to their effectiveness at low concentrations, relatively low mammalian toxicity, and relative cost. However, the extensive use and over-reliance of pyrethroids for vector control has resulted in and raised major concerns over the development of resistance [6–8]. Physiological resistance to pyrethroids is now widespread in the major malaria vectors in Africa [9] and in dengue vectors in Southeast Asia [10] as this is the only class of insecticide available for use on ITN/LLINs. This resistance has reduced the efficacy of pyrethroids which consequently has increased mosquito survival which is a prelude to a rising incidence of malaria and dengue [2]. Moreover, conventional methods of control such IRS and ITNs are not suitable for protection against outdoor transmission and there are few alternatives to effectively combat aspect of exposure to infection [11].

*Aedes aegypti* (L.) and *Aedes albopictus* (Skuse) are the two most important vectors of dengue as well as chikungunya and Zika viruses [1]. In general, both species are found commonly throughout Thailand and can occupy a broad range of indoor or outdoor larval habitats [12]. *Aedes aegypti*, in particular, is a formidable mosquito to control using current vector abatement methods. It is a strongly synanthropic/anthropophilic species and predominantly found in and near human dwellings with a high tendency of adults to rest inside houses [13].

Among the primary *Anopheles* malaria vector species in Thailand, *Anopheles dirus* Peyton & Harrison and *An*. *minimus* Theobald are among the most important [14, 15]. Both species are commonly found along the Thai-Myanmar and Thai-Cambodia international borders and responsible for the majority of transmission [16]. Although their relative importance as vectors may vary depending on locality, season, and epidemiological circumstances, both species exhibit exophagic (outdoor biting) behavior, thus complicating control efforts [16, 17]. Preferential early evening and morning outdoor biting [11, 14, 18] increases the risk of transmission where people are not properly protected by conventional vector control tools (e.g., ITNs and IRS) or lack of personal protection tools (e.g., topical repellents, long clothing, insecticide-treated clothing) [19].

Therefore, there has been increased interest in the spatial repellent properties of certain AIs as a possible means of arresting outdoor biting [20–22]. By disrupting or interfering the normal behavioral patterns of vector host-seeking and biting, the vapor-phase properties of chemicals can potentially create a spatial ‘vector-free space’ and thus reduce the risk of so-called ‘residual’ transmission as well as indoor [20, 23]. Transfluthrin (TFT) is a relatively recent volatile AI pyrethroid that has been incorporated in a variety of commercial products (i.e., mosquito coils, aerosol sprays) and has been explored for promoting spatial repellent as a new invention class against mosquitoes [21, 22, 24]. Estrada et al. [25] demonstrated that TFT produced the strongest spatial repellency response in *Ae*. *aegypti* (37.5% repellency) at 0.001% and *Ae*. *albopictus* (45% repellency) at 0.01%. However, to our knowledge, there have been no published studies with TFT distinguishing the two types of behavioral avoidance responses (irritancy and repellency). Information on TFT and mosquito responses are limited; therefore, investigations are required on this AI for development of innovative spatial repellent products.

A better understanding of the functional effects of chemical insecticides and mosquito responses is, therefore, critical in designing practical interventions of TFT-based spatial repellent products [20, 26]. The three primary modes of action of a chemical are insecticidal (killing) and two behavioral responses, either by contact locomotor excitation (‘irritancy’) and/or noncontact spatial repellency [26]. The excito-repellency (ER) assay system is a well-established behavioral testing system to evaluate sublethal chemical actions such as contact excitation and noncontact repellency of synthetic and natural-derived compounds [27–36]. By measuring flight movement, i.e. ‘escape’ responses, the assay measures the degree of excitation or repellency exerted by chemicals on mosquitoes. The ER assay is a suitable bioassay system to evaluate the functional properties involving behavioral avoidance actions of TFT against important mosquito vectors. In the present study, the dose-response escape movement of three laboratories and two field-collected mosquito populations, exposed to species-specific sublethal, lethal and discriminating concentrations of TFT are described herein. These results, using this innovative assay, will provide researchers and product development groups important information to accelerate access to new and innovative tools to combat difficult and persistent mosquito-borne diseases [20].

## Materials and methods

### Mosquitoes

Three pyrethroid-susceptible laboratory strains and two recently collected field populations of adult mosquitoes were used. Laboratory strains: 1) *Aedes aegypti* (L.) (USDA strain), obtained from the U.S. Department of Agriculture, was maintained at the Department of Entomology, Faculty of Agriculture, Kasetsart University (KU) for over 20 years. 2) *Anopheles minimus* s.s. Theobald (DDC strain), from the Malaria Division, Department of Disease Control, Ministry of Public Health, Nonthaburi, Thailand, originally collected in Rong Klang District, Prae Province, northern Thailand in 1993 and maintained at the Kasetsart University laboratory shortly thereafter. 3) *Anopheles dirus* s.s. Peyton & Harrison (TMMU strain) was originally collected in Khao Mai Kaeo Sub-district, Bang Lamung District, Chonburi Province, eastern Thailand in 1981 and obtained from the Department of Medical Entomology, Faculty of Tropical Medicine, Mahidol University in 2016. This strain has been maintained continuously at KU insectary since 2016. Mosquito colonies were reared following standard handling procedures and conditions (25±2°C, 80±10% relative humidity and 12:12 h light:dark cycle) at the KU laboratory [28, 30]. Larval food (TetraMin®, TetraGmbH, Germany) were provided 3 times daily. Each day collected pupae were placed in small holding cups and adults were allowed to emerge in wire-mesh cages (30 x 30 x 30 cm) and provided *ad libitum* with 10% sucrose (w/v) solution as sustenance. An artificial membrane feeding technique [37] using human whole blood was used to maintain the mosquito colonies. The pathogen-free blood was provided from The Thai Red Cross Society and handled in the KU insectary following a written standard operating procedure. An artificial insemination technique [38] was required for continuous colonization of *An*. *dirus*; all other species were self-mating in holding cages.

Field populations: 1) *Aedes aegypti* field population was collected as immature stages from Muang District, Nonthaburi Province, central Thailand (13°50’N; 100°29’E) in 2016 and maintained at KU insectary; 2) *Anopheles harrisoni* Harbach & Manguin (a species member of the Minimus Complex) field population was collected from Pu Teuy Village, Sai Yok District, Kanchanaburi Province (14°20’N; 98°59’E) according to Sungvornyothin et al. [17]. The assessing of mosquito collection site was approved by the Division of Agriculture and Cooperatives, Office of Military Development, Armed Forces Development Command, Royal Thai Armed Forces. All wild-caught anophelines were initially identified using morphological keys [39] prior to testing. Molecular identification was subsequently applied to confirm species thereafter [17]. All ER tests included F_1_ to F_3_ generation mosquitoes.

### Insecticide

Technical grade TFT (2,3,5,6-tetrafluorobenzyl (1*R*,3*S*)-3-(2,2-dichlorovinyl)-2,2-dimethylcyclopropanecarboxylate; 97.90% purity) was used. Transfluthrin stock solutions were prepared using analytical grade acetone (Baker Analyzed® A.C.S. reagent) as an organic solvent and silicone oil (Dow Corning® 556 cosmetic grade) as a carrier at a ratio of 1.91:0.95.

### Insecticide impregnated papers

Whatman No. 1 filter papers, measuring 14.7 x 17.5 cm, were uniformly impregnated with 2.8 mL of TFT solution using a calibrated micropipette. Papers were treated with established 50%, 75% and 99% lethal concentrations (LC_50_, LC_75_ and LC_99_) and a discriminating concentration (DC) based on doubling the LC_99_ x 2 for *Ae*. *aegypti*, *An*. *minimus* and *An*. *dirus* [40], respectively (Tables 1–3). Untreated control papers were prepared with acetone and silicone oil only. Treated papers were air-dried at room temperature on the aluminium foil for 24 h before use. The ambient conditions and drying time matched a previous study [40] to achieve approximately same percent concentration at time of test. Each prepared paper was used only once and discarded.

**Table 1:** Mean percent escape response and percent mortality of *Ae*. *aegypti* (USDA) exposed to TFT in ER assays.

% Concentration†	ER assay	Percent escape response				Percent knockdown and mortality in treatments***			
		Control		Treatment		% KD 30-min exposure		% Mortality 24 h	
		N	%Esc.*	N	%Esc.**^,(^^§^^)^	Esc.	NEsc.	Esc.	NEsc.
0.00852 (LC_50_)	Noncontact	60	0.0^a^	60	5.00^a^	0.0^a^	0.0^a^	0.0^a^	5.26^a^
	Contact	60	0.0^a^	60	48.33^b^ (43.33)	0.0^a^	25.81^b^	3.45^a^	6.45^a^
0.01274 (LC_75_)	Noncontact	60	6.67^a^	59	29.18^a^	0.0^a^	0.0^a^	0.0^a^	2.56^a^
	Contact	60	1.67^a^	58	60.34^b^ (26.45)	5.71^a^	47.83^b^	2.86^a^	8.70^a^
0.03412 (LC_99_)	Noncontact	63	6.35^a^	59	23.98^a^	0.0^a^	0.0^a^	0.0^a^	0.0^a^
	Contact	60	8.33^a^	61	66.02^b^ (40.04)	4.76^a^	94.74^b^	2.38^a^	68.42^b^
0.06824 (DC)	Noncontact	60	5.00^a^	59	28.63^a^	36.84^a^	82.50^a^	0.0^a^	55.00^a^
	Contact	60	6.67^a^	60	37.50^a^ (9.46)	24.00^a^	85.71^a^	4.00^a^	31.43^a^

ER, excito-repellency; Esc, escaped mosquitoes; NEsc, non-escaped mosquitoes; KD, knockdown; LC, lethal concentration; DC, discrinimating concentration.

*Percent escape in control group showed no significantly difference (*P* > 0.05) between noncontact and contact tests at the same concentration.

**Percent escape adjusted with paired controls using Abbott’s formula. Different letter indicates significant differences (*P* < 0.05) between noncontact and contact tests in treatment group at the same concentration.

***Different letter indicates significant differences (*P* < 0.05) between control, noncontact and contact tests at the same concentration and same response group (Esc. or NEsc.).

^**§**^Percent contact escape adjusted with paired noncontact escape response using modified Henderson-Tilton’s formula.

†Due to TFT vaporization during drying period, the actual percent concentration at the time of testing is less than initial paper treatment percent indicated.

30-min KD and 24-hr mortality not observed in controls.

**Table 2:** Mean percent escape response and percent mortality of *An*. *minimus* (DDC) exposed to TFT in ER assays.

% Concentration†	ER assay	Percent escape response				Percent knockdown and mortality in treatments***			
		Control		Treatment		% KD 30-min exposure		% Mortality 24 h	
		N	%Esc.*	N	%Esc.**^,(^^§^^)^	Esc.	NEsc.	Esc.	NEsc.
0.00423 (LC_50_)	Noncontact	60	15.00^a^	64	60.07^a^	11.11^a^	26.32^a^	2.22^a^	0.0a
	Contact	61	6.56^a^	60	85.73^a^ (16.35)	1.92^b^	37.50^a^	0.0^a^	12.50^a^
0.00760 (LC_75_)	Noncontact	60	11.67^a^	54	66.46^a^	18.42^a^	62.50^a^	0.0^a^	12.50^a^
	Contact	59	10.17^a^	58	80.81^a^ (12.38)	10.42^a^	80.00^a^	4.17^a^	60.00^b^
0.03191 (LC_99_)	Noncontact	60	5.00^a^	58	54.63^a^	63.64^a^	80.00^a^	0.0^a^	12.00^a^
	Contact	60	8.33^a^	62	52.49^a^ (0)	65.71^a^	100.0^a^	2.86^a^	55.56^b^
0.06382 (DC)	Noncontact	60	15.00^a^	60	58.82^a^	71.79^a^	71.43^a^	2.56^a^	14.29^a^
	Contact	60	11.67^a^	60	43.39^a^ (0)	83.33^a^	96.67^b^	26.67^b^	50.00^b^

ER, excito-repellency; Esc, escaped mosquitoes; NEsc, non-escaped mosquitoes; KD, knockdown; LC, lethal concentration; DC, discrinimating concentration.

*Percent escape in control group showed no significantly difference (*P* > 0.05) between noncontact and contact tests at the same concentration.

**Percent escape adjusted with paired controls using Abbott’s formula. Different letter indicates significant differences (*P* < 0.05) between noncontact and contact tests in treatment group at the same concentration.

***Different letter indicates significant differences (*P* < 0.05) between control, noncontact and contact tests at the same concentration and same response group (Esc. or NEsc.).

^**§**^Percent contact escape adjusted with paired noncontact escape response using modified Henderson-Tilton’s formula.

†Due to TFT vaporization during drying period, the actual percent concentration at the time of testing is less than initial paper treatment percent indicated.

30-min KD and 24-hr mortality not observed in controls.

**Table 3:** Mean percent escape response and percent mortality of *An*. *dirus* (TMMU) exposed to TFT in ER assays.

% Concentration†	ER assay	Percent escape response				Percent knockdown and mortality in treatments***			
		Control		Treatment		% KD 30 min exposure		% Mortality 24 h	
		N	%Esc.*	N	%Esc.**^,(^^§^^)^	Esc.	NEsc.	Esc.	NEsc.
0.00409 (LC_50_)	Noncontact	60	8.33^a^	60	80.00^a^	75.51^a^	18.18^a^	14.29^a^	63.64^a^
	Contact	60	10.00^a^	60	74.08^a^ (0)	78.26^a^	100.0^b^	32.61^a^	50.00^a^
0.00489 (LC_75_)	Noncontact	62	12.90^a^	60	46.42^a^	93.75^a^	96.43^a^	40.63^a^	89.29^a^
	Contact	61	9.84^a^	60	59.33^a^ (10.00)	73.68^a^	100.0^a^	31.58^a^	90.91^a^
0.00754 (LC_99_)	Noncontact	60	8.33^a^	62	85.93^a^	40.74^a^	100.0^a^	3.70^a^	37.50^a^
	Contact	62	4.84^a^	62	64.52^b^ (0)	65.00^a^	100.0^a^	2.50^a^	50.00^a^
0.01508 (DC)	Noncontact	60	15.00^a^	60	49.02^a^	94.12^a^	100.0^a^	50.00^a^	100.0^a^
	Contact	62	9.68^a^	58	54.19^a^ (1.95)	100.0^a^	100.0^a^	55.88^a^	95.83^a^

ER, excito-repellency; Esc, escaped mosquitoes; NEsc, non-escaped mosquitoes; KD, knockdown; LC, lethal concentration; DC, discrinimating concentration.

*Percent escape in control group showed no significantly difference (*P* > 0.05) between noncontact and contact tests at the same concentration.

**Percent escape adjusted with paired controls using Abbott’s formula. Different letter indicates significant differences (*P* < 0.05) between noncontact and contact tests in treatment group at the same concentration.

***Different letter indicates significant differences (*P* < 0.05) between control, noncontact and contact tests at the same concentration and same response group (Esc. or NEsc.).

^**§**^Percent contact escape adjusted with paired noncontact escape response using modified Henderson-Tilton’s formula.

†Due to TFT vaporization during drying period, the actual percent concentration at the time of testing is less than initial paper treatment percent indicated.

30-min KD and 24-hr mortality not observed in controls.

### Excito-repellency (ER) assay system

Each test trial consisted of four identical ER chambers as described previously [41]. For measuring spatial repellency, a pair of noncontact chambers, one containing TFT treated papers and the other fitted with untreated control papers is designed so that mosquitoes are unable to make direct physical contact with the treated surface by placement of a screen mesh barrier separating the inner chamber and chamber wall with the paper. For the contact design, paired treatment and control chambers excluded the mesh barrier, thus allowing mosquitoes to make free contact with the impregnated papers. The ER system is windowless, thus preventing observation of mosquito behavior inside the chamber during the testing period. The only light entering the chamber is via the open exit portal. Primary outcome measures are escaped by flight from the chamber, and the knockdown and mortality response of mosquitoes that exit the chamber and those that remine after 30 min. Higher in percent escape would indicate the greater degree in repellent property, either by contact or noncontact, of the tested compound.

Before beginning a trial, all test mosquitoes are deprived a sugar meal for 24 h before testing and provided only water on cotton pads. At the time of test, 15 non-blood-fed, 3–5 day-old female mosquitoes (single species) were introduced into each of four chambers (total 60 mosquitoes per complete trial run). Following a 3-min ‘acclimation’ period, the exit portal was opened for each chamber to initiate observations (escape). The number of mosquitoes that escaped from each exposure chamber into the attached receiving cage were recorded at 1-min intervals for a period of 30 min. At post-exposure, all mosquitoes were transferred to holding cups labeled by the chamber and movement response (escape or non-escape). The number of dead or knockdown (moribund) mosquitoes were recorded separately from inside (those remaining) and outside (having escaped) each chamber. All live and moribund mosquitoes in the chamber were held separately and provided with cotton-soaked 10% sugar solution for 24-h before recording the final mortality determination. The ambient air temperatures and relative humidity were recorded at the beginning of each 30-min trial period. All trials were performed during daytime hours (09:00–16:30 h) and each trial series were replicated four times. All assays were carried out in the same laboratory space, under identical conditions and ER assay systems for both colonized and field-collected mosquitoes.

## Data analysis

The percent ER escape responses and 24-h mortality were adjusted using Abbott’s formula when escape or mortality in the paired controls (untreated chambers) was between 5 and 20% of the test sample [42]. The number of mosquitoes escaping from the contact chamber is potentially a measure of the combined action of contact excitation and spatial repellency. Therefore, the data was subjected to a second adjustment to estimate escape activity resulting from contact alone using the simultaneous paired noncontact escape response. The following equation was used to account for any unequal sample sizes between pairings: (1 - [number of contact in test x number of noncontact escape / number of noncontact in test x number of contact escape]) x 100. This equation, the reciprocal of the Henderson-Tilton (H-T) formula [43], compares the escape response in paired contact and noncontact chambers to exclude the repellency effect inside the contact chamber and provide an estimate of the ‘true’ contact effect [30].

Using initial escape data (Abbott’s formula adjusted only), Kaplan-Meier survival analysis was used to estimate rate of mosquito escape in contact and noncontact test formats, and for comparing differences in mosquito escape between the four concentrations [44]. The time in minutes for 25% (ET_25_), 50% (ET_50_) and 75% (ET_75_) of test population to escape was estimated. A log-rank test [45] compared patterns of behavioral escape within species and between concentrations using SAS 6.10 (SAS Institute, Cary, NC). The percent knockdown and mortality between noncontact and contact tests in treatment group at the same concentration were compared using Mann-Whitney U test. All statistical significance was set at 5% (*P* < 0.05).

## Results

### Excito-repellency of colonized mosquitoes to TFT

**Fig 1:**
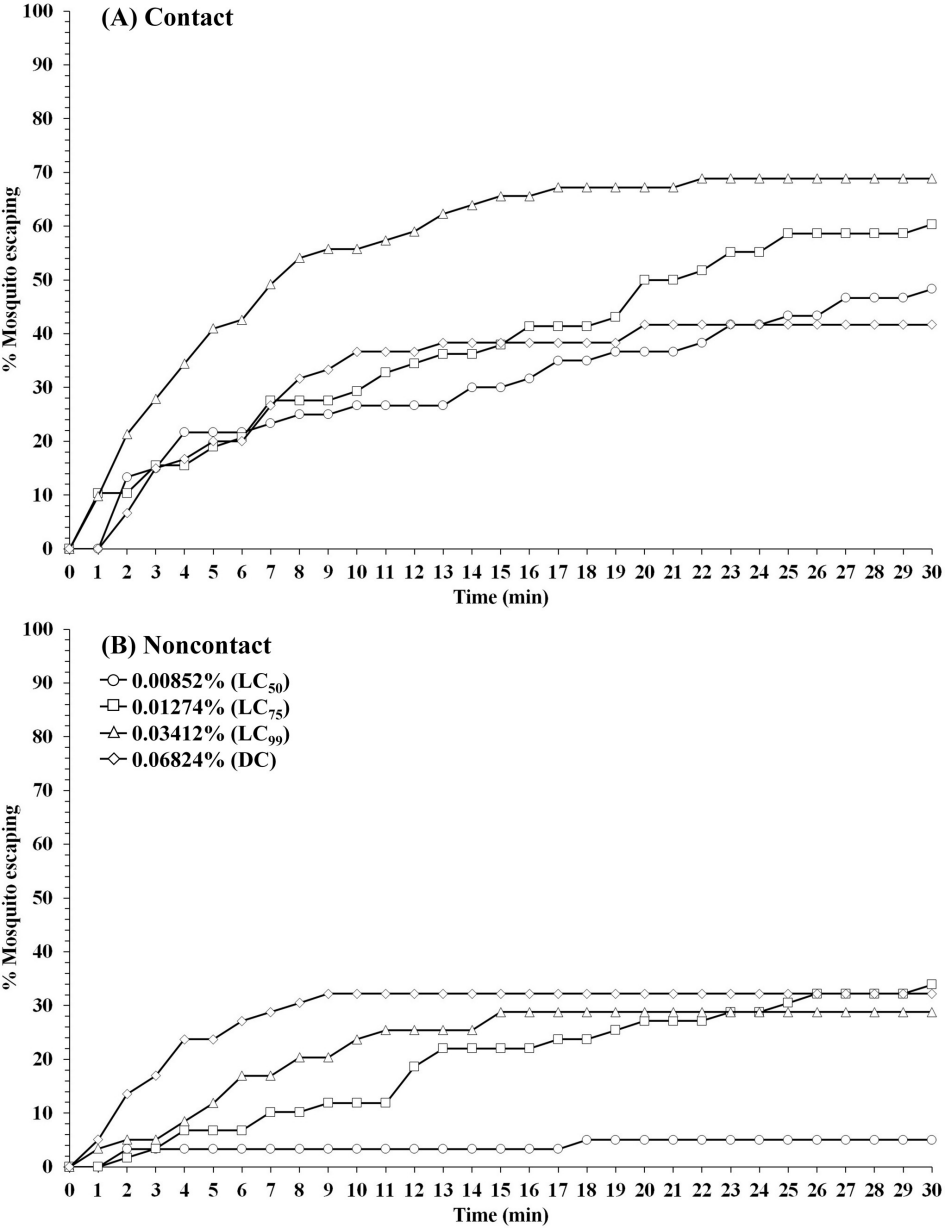
Patterns of percentage escaping for *Ae*. *aegypti* laboratory strain (USDA) in (A) contact and (B) noncontact ER assays. Escape responses recorded at 1-min intervals for 30-min exposure to TFT at 0.00852% (LC_50_), 0.01274% (LC_75_), 0.03412% (LC_99_), and 0.06824% (DC). Paired control escape responses not shown.

**Fig 2:**
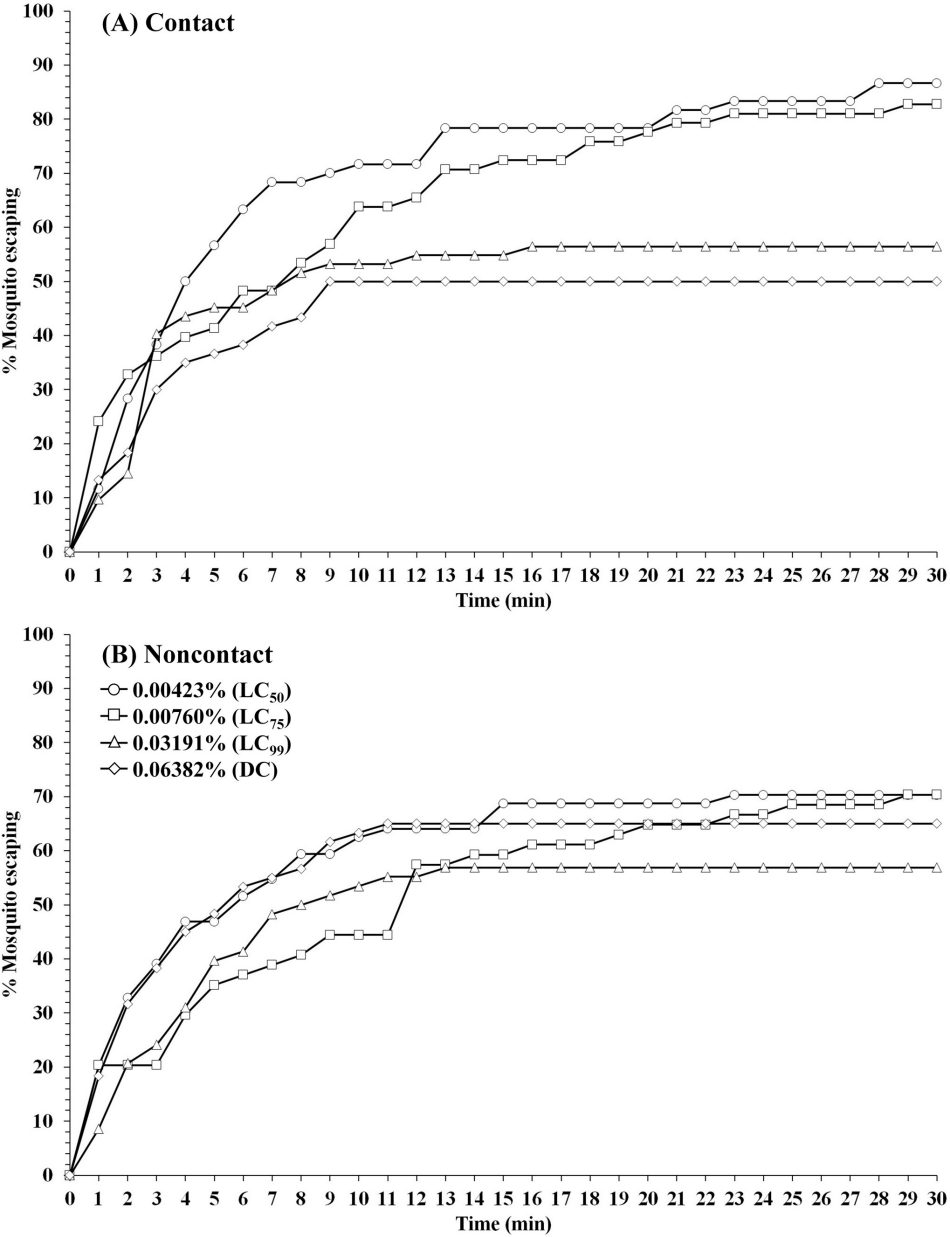
Patterns of percentage escaping for *An*. *minimus* laboratory strain (DDC) in (A) contact and (B) noncontact ER assays. Escape responses recorded at 1-min intervals for 30-min exposure to TFT at 0.00423% (LC_50_), 0.0076% (LC_75_), 0.03191% (LC_99_), and 0.06382% (DC). Paired control escape responses not shown.

**Fig 3:**
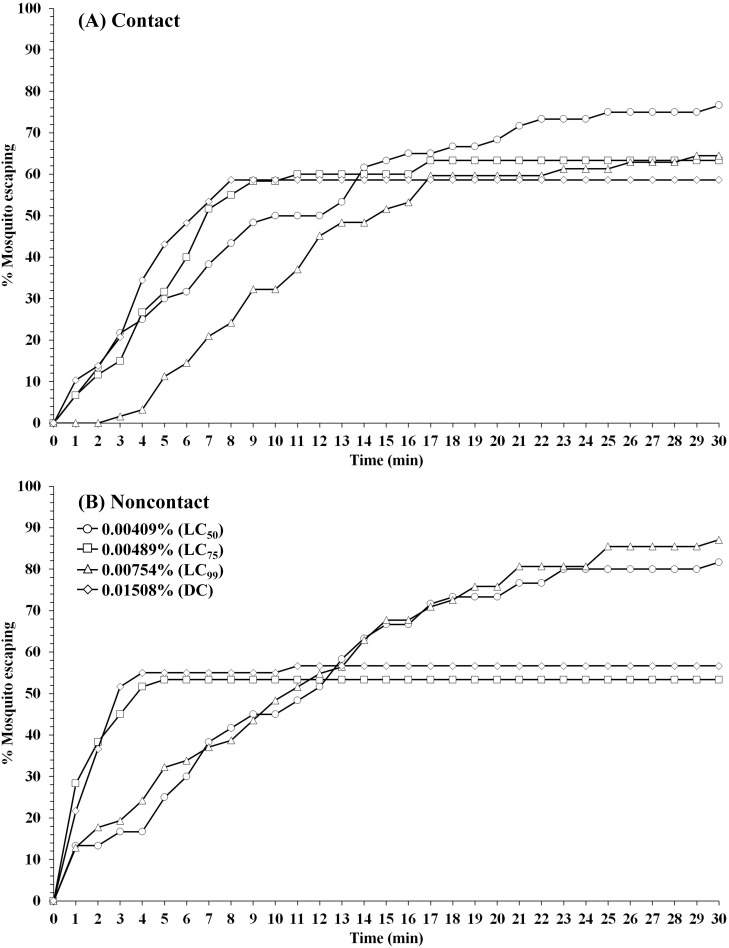
Patterns of percentage escaping for *An*. *dirus* laboratory strain (TMMU) in (A) contact and (B) noncontact ER assays. Escape responses recorded at 1-min intervals for 30-min exposure to TFT at 0.00409% (LC_50_), 0.00489% (LC_75_), 0.00754% (LC_99_), and 0.01508% (DC). Paired control escape responses not shown.

The contact excitation and noncontact repellency effects of TFT on laboratory-reared *Ae*. *aegypti*, *An*. *minimus* and *An*. *dirus* using different species-specific concentrations are shown in Tables 1–3 and Figs 1–3. Overall, mosquitoes exposed to TFT had significantly (*P* < 0.05) greater percent escape in both noncontact and contact trials compared to the controls, except for *Ae*. *aegypti* in noncontact at LC_50_ (*P* = 0.0807) (S1 Table). No significant different in percent escape was found between noncontact and contact trial in the control group at the same concentration (*P* > 0.05) (S2 Table). For *Ae*. *aegypti* (USDA), LC_50_ to LC_99_ produced percent escape in contact tests (Abbott’s adjusted percent only) ranging between 48 and 66% (Table 1). The percent escape in contact tests was inverse between the lower concentrations (0.00852% to 0.01274%), and LC_99_ (0.03412%) and the DC (0.06824%) due to the higher knockdown (KD) effect inside the chamber (25.8–47.8% vs. 85.7–94.7% KD for LC_50_-LC75 and LC_99_-DC, respectively) compared to those that escaped (4.8–24% KD). Conversely, a positive relationship was found in noncontact test as percent escape is increasec as TFT concentration increased (Tables 1 and 4).

**Table 4:** Time in minutes for 25% (ET_25_), 50% (ET_50_), and 75% (ET_75_) of mosquitoes to escape treated chambers containing different TFT concentrations.

Mosquito species	Concentration (%)	Noncontact (min)			Contact (min)		
		ET_25_	ET_50_	ET_75_	ET_25_	ET_50_	ET_75_
*Ae*. *aegypti*	0.00852 (LC_50_)	-	-	-	8	-	-
(USDA)	0.01274 (LC_75_)	19	-	-	7	20	-
	0.03412 (LC_99_)	11	-	-	3	8	-
	0.06824 (DC)	6	-	-	7	-	-
*An*. *minimus*	0.00423 (LC_50_)	2	6	-	2	5	13
(DDC)	0.00760 (LC_75_)	5	12	-	2	8	18
	0.03191 (LC_99_)	4	9	-	3	8	-
	0.06382 (DC)	2	6	-	3	9	-
*An*. *dirus*	0.00409 (LC_50_)	5	12	21	4	10	25
(TMMU)	0.00489 (LC_75_)	1	4	-	4	7	-
	0.00754 (LC_99_)	5	11	19	9	15	-
	0.01508 (DC)	2	3	-	4	7	-

(-) Not applicable as too few mosquitoes escaped from chambers precluding escape time (ET) calculation.

LC, lethal concentration; DC, discriminating concentration.

For *An*. *minimus* (DDC), LC_50_ and LC_75_ TFT exhibited the highest mean percent escape in both contact and noncontact tests, ranging from 85.7–80.8% (contact) and 60.1–66.5% (noncontact), respectively (Table 2). For contact tests, final percent escape was inverse to increasing concentration, 85.7% at LC_50_ and 43.4% at the highest concentration (DC). Relatively high repellent activity in noncontact test was observed with *An*. *dirus* (TMMU) ranging from 46.4% escape (LC_75_) to 85.9% escape (LC_99_), while the contact test showed a final percent escape ranging between 54.2% (DC) and 74.1% (LC_50_) across all four concentrations (Table 3).

A wide range of escape time (ET) activity was observed between species and concentrations in noncontact and contact tests (Table 4). In only a few instances did 50% or more of mosquitoes escape the chambers within 30 min. For contact tests, the ET_75_ values for *An*. *minimus* were 13 and 18 min at LC_50_ and LC_75_, respectively, compared to 6 and 12 min for ET_50_ values in noncontact tests. Only at LC_50_ that 75% of *An*. *dirus* escaped from both noncontact (ET_75_ = 21 min) and contact (ET_75_ = 25 min) tests. Overall, at all concentrations, at least 50% of *An*. *minimus* and *An*. *dirus* escaped TFT-treated chambers in both trials within 6–12 and 3–15 min (ET_50_), respectively. In contrast, *Ae*. *aegypti* showed the least excito-repellency, both in final lesser percent escape and longer length of time required to escape the chamber.

For *Ae*. *aegypti*, unadjusted contact escape was significantly stronger than noncontact repellency between LC_50_ and LC_99_, but not the DC (Table 1). For both *Anopheles* species, nearly all pairings from LC_50_ to DC showed no significant difference in final percent escape between contact and noncontact, except for *An*. *dirus* at LC_99_ showing a significantly stronger repellency response (Tables 2 and 3).

High percentage of knockdown (82.5–94.7%) was only observed against *Ae*. *aegypti* at high concentration (LC_99_ and DC) in non-escape mosquito, while no knockdown (0%) and low to moderate %KD (4.8–36.8%) was found in escape group for all concentration in both contact and noncontact tests (Table 1). For *An*. *minimus*, moderate to high %KD was found at LC_75_-DC and LC_99_-DC for non-escape (62.5–100%) and escape (63.6–83.3%) mosquito, respectively (Table 2). Whereas, relatively high %KD was found in nearly all concentrations for non-escape (96.4–100%) and escape (73.7–100%) *An*. *dirus* in both ER assays (Table 3). However, test mosquitoes were able to recover within 24 h as evidenced in reduced % mortality in nearly all treatments for both non-escape and escape group (Tables 1–3).

Using first round analysis adjustments only (Abbott’s formula) for escape data, the survival curves at 1-min intervals in contact and noncontact tests were generated (Figs 1–3). Comapred to the two anopheline species, *Ae*. *aegypti* had relatively lower escape rates in noncontact tests at all concentrations, and more moderate responses in the contact tests (Fig 1). Conversely, both *Anopheles* species showed similarly greater overall escape and relatively more rapid exit times in both test exposure formats (Figs 2 and 3).

An estimation of ‘true’ contact excitation was calculated by using a second round analytical adjustment of the final percent contact escape against the paired percent noncontact escape (Tables 1–3). After adjustment, *Ae*. *aegypti* exhibited only moderate behavioral avoidance (between 26.5–43.3% escape) with contact exposure at LC_50_—LC_99_, and only contributed 9.5% of the escape at the highest concentration (DC) (Table 1). However, the second round estimates revealed much lower contact effects (16.4 and 12.4% escape) at LC_50_ and LC_75_, repectively, for *An*. *minimus* (Table 2) and 10% escape or less at all concentrations for *An*. *dirus* (Table 3). These findings suggest an overall minor contribution of physical contact with TFT compared to spatial repellency producing behavioral responses in the two colonized anophelines (Tables 1–3).

Within species, log-rank multiple paired comparisons between TFT concentrations and escape responses in contact and noncontact tests were performed (Table 5). In contact tests, *Ae*. *aegypti* showed no significant differences in escape responses except for LC_50_- LC_99_ and LC_99_-DC pairings. For noncontact tests, all LC_50_ pairings with the other three higher concentration were significantly different. For *An*. *minimus*, significant differences in contact escape were found in four pairwise comparisons (excluding LC_50_-LC_75_ and LC_99_-DC); whereas there was no difference in escape between noncontact comparisons. For *An*. *dirus*, no significant differences were observed in contact escape pairings and in only one instance was a difference detected in the noncontact assays (LC_75_-LC_99_).

**Table 5:** Pairwise log-rank comparisons of mosquito escape responses between various transfluthin concentrations in noncontact and contact ER assays by species.

Concentration	*Ae*. *aegypti* (USDA)		*An*. *minimus* (DDC)		*An*. *dirus* (TMMU)	
(%)	Noncontact	Contact	Noncontact	Contact	Noncontact	Contact
LC_50_ vs LC_75_	<0.0001*	0.2003	0.4407	0.4442	0.1181	0.4636
LC_50_ vs LC_99_	0.0006*	0.0041*	0.1073	0.0021*	0.5945	0.0617
LC_50_ vs DC	0.0001*	0.6230	0.6849	0.0002*	0.2464	0.3553
LC_75_ vs LC_99_	0.7607	0.0689	0.4071	0.0122*	0.0274*	0.2316
LC_75_ vs DC	0.7831	0.1126	0.7244	0.0021*	0.8270	0.9979
LC_99_ vs DC	0.5229	0.0015*	0.2351	0.5263	0.0722	0.3403

(*) significant difference at 95% CI; LC, lethal concentration; DC, discriminating concentration.

### Excito-repellency of field-collected mosquitoes to sublethal concentration

WHO insecticide susceptibility tests showed that field-collected *Ae*. *aegypti* (NON) were found resistant to TFT (< 56% mortality at 24-h) using the previously established DC (0.06824%); whereas, *An*. *harrisoni* (KAN) was fully susceptible to TFT using the DC 0.06382% [40].

**Fig 4:**
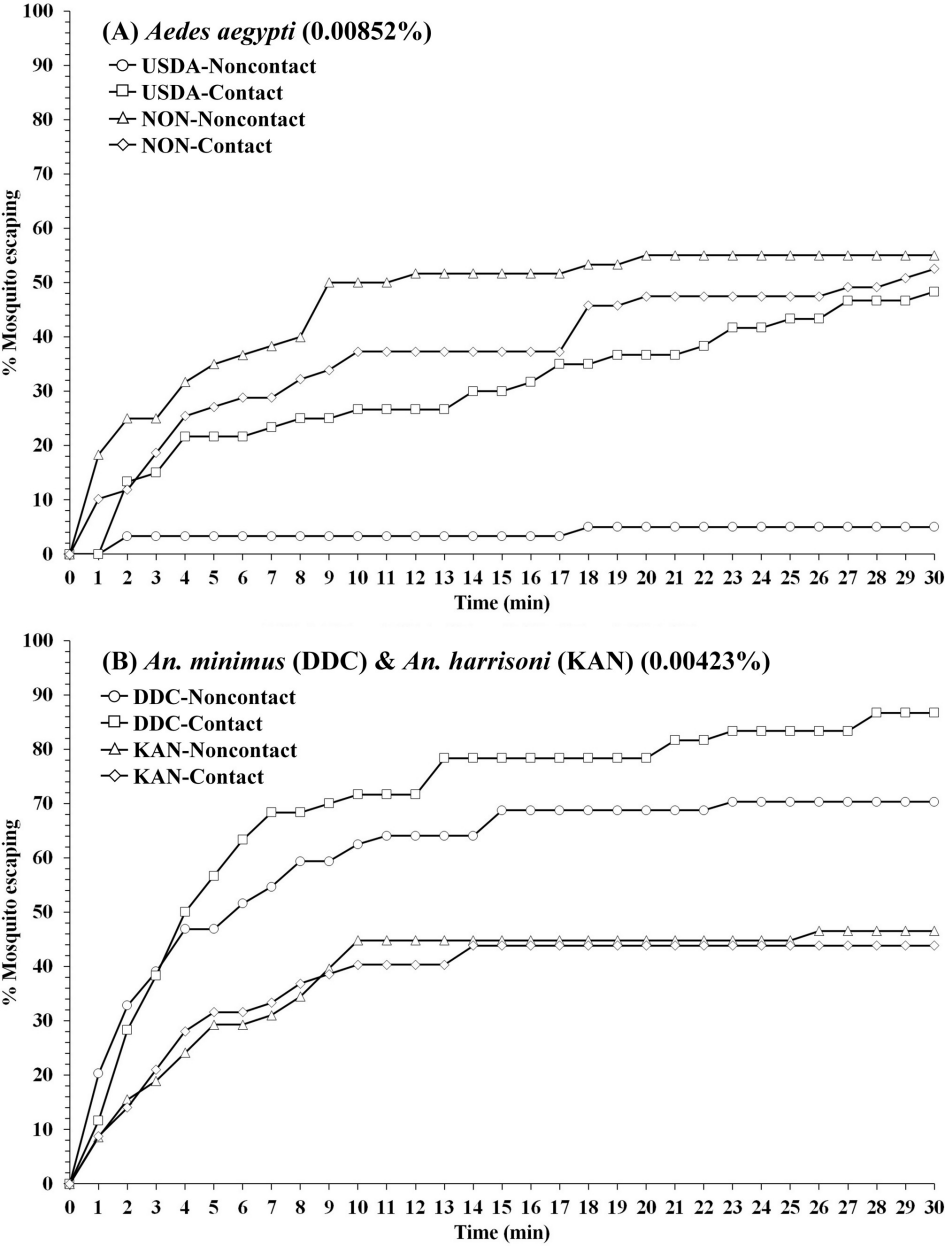
Comparison percentage escape patterns for (A) *Ae*. *aegypti* USDA strain and Nonthaburi (NON) field population, and (B) *An*. *minimus* DDC strain and *An*. *harrisoni* Kanchanaburi (KAN) field population in contact and noncontact ER assays. Escape responses recorded at 1-min intervals during 30-min exposure to TFT at LC_50_ established for each laboratory strain [40]. Concentration for *An*. *minimus* applied for *An*. *harrisoni*. Paired control escape responses not shown.

A single sublethal TFT concentration (LC_50_) established for *Ae*. *aegypti* (USDA) (0.00852%) and *An*. *minimus* (DDC) (0.00423%) [41] were used for measuring escape response of *Ae aegypti* (NON) and *An*. *harrisoni* (KAN), respectively. In noncontact tests, *Ae*. *aegypti* (NON) had significantly higher escape response compared to the USDA colony (*P* < 0.0001; Fig 4, Tables 1 and 6), while there was no significant difference in contact tests (*P* = 0.5395). There was a significantly higher percent escape in *An*. *minimus* (DDC) compared to *An*. *harrisoni* (KAN) in both noncontact (*P* = 0.0048) and contact (*P* < 0.0001) tests (Fig 4, Tables 2 and 6). For *An*. *harrisoni*, approximately 30% and 40.6% escape were observed in the contact and noncontact tests, respectively, far lower than seen with colonized *An*. *minimus* (85.7% contact, 60% noncontact) (Table 6). Following a second round adjustment of percent contact escape, only spatial repellency was apparent against both field populations. After adjustment, significant (*P* < 0.0001) reductions in estimated percent escape due to contact only was also seen in the colonized strains. High % knockdown (>93.6%) were also observed in non-escape *An*. *harrisoni* KAN for both contact and noncontact tests, while 68–74% knockdown was found for non-escape *Ae*. *aegypti* NON mosquito. Both field populations recovered (reduce % mortality) after 24-h post exposure with reduced mortality ranging from 0% to 20% (Table 6).

**Table 6:** Mean percent escape response and percent mortality of *Ae*. *aegypti* and *An*. *harrisoni* field populations exposed to LC_50_ TFT.

Species	ER assay	Percent escape response				Percent knockdown and mortality in treatments***			
(% Concentration)†		Control		Treatment		% KD 30-min exposure		% Mortality 24 h	
		N	%Esc.*	N	%Esc.**^,(^^§^^)^	Esc.	NEsc.	Esc.	NEsc.
*Ae*. *aegypti* (NON)	Noncontact	59	1.69^a^	60	55.00^a^	3.03^a^	74.07^a^	0.0^a^	25.93^a^
(0.00852)	Contact	60	3.33^a^	59	52.54^a^ (0)	0.0^a^	67.86^a^	3.23^a^	17.86^a^
*An*. *harrisoni* (KAN)	Noncontact	60	10.00^a^	58	40.61^a^	51.85^a^	93.55^a^	7.41^a^	74.19^a^
(0.00423)	Contact	60	20.00^a^	57	29.83^b^ (0)	40.00^a^	100.0^a^	20.00^a^	75.00^a^

ER, excito-repellency; Esc, escaped mosquitoes; NEsc, non-escaped mosquitoes; KD, knockdown.

*Percent escape in control group showed no significantly difference (*P* > 0.05) between noncontact and contact tests at the same concentration.

**Percent escape adjusted with paired controls using Abbott’s formula. Different letter indicates significant differences (*P* < 0.05) between noncontact and contact tests in treatment group at the same concentration.

***Different letter indicates significant differences (*P* < 0.05) between control, noncontact and contact tests at the same concentration and same response group (Esc. or NEsc.).

^**§**^Percent contact escape adjusted with paired noncontact escape response using modified Henderson-Tilton’s formula.

†Due to TFT vaporization during drying period, the actual percent concentration at the time of testing is less than initial paper treatment percent indicated.

30-min KD and 24-hr mortality not observed in controls.

## Discussion

The key feature of the excito-repellency (ER) assay system is the separation between contact excitation (‘irritancy’) and noncontact spatial repellency of a target compound that result in movement away or non movement from the physical source of the active compound (i.e. impregnated papers) [27,44]. Using this system, we measured the behavioral response (escape from the treated chamber) of TFT against colonized strains of *Ae*. *aegypti*, *An*. *minimus* and *An*. *dirus*. When allowing mosquitoes a free flight choice, TFT was most effective in eliciting substantial spatial repellency, particularly *Anopheles* mosquitoes. Relatively high escape reactions in both contact and noncontact tests with all concentrations, lethal and sublethal, occurred with *An*. *minimus* and *An*. *dirus*. *Aedes aegypti* showed a more reduced and delayed escape responses compared to the anophelines. Transfluthrin at LC_50_, LC_75_, and LC_99_ also induced a moderate knockdown effect and 24 h mortality against *Ae*. *aegypti*, but higher KD and mortality on anophilines, particularly *An*. *dirus*. At the final concentrations used, the lower post-exposure (24 h) mortality compared with the higher initial percentage of knockdown mosquitoes at 30 min (i.e., recovery over time), for those not escaping (remaining in the exposure chamber) and those successfully escaping the chambers, suggests that the overall behavioral avoidance was the primary mode of action of TFT.

Previously, the standard WHO susceptibility assay, using lethal and discriminating concentrations of TFT on cellulose filter papers, was used to measure phenotypic response of mosquitoes with survival or mortality as endpoints [40]. Given that TFT is highly volatile and its bioavailability on cellulose papers is time-dependent, we acknowledge that the treatment of filter papers followed by a 24-h period of air drying at room temperature before testing poses a potential, if not significant, problem by not reporting the actual concentrations used at time of testing [40]. This study reports the initial concentration applied on the papers; however, we did not attempt to chemically determine the amount of active ingredient remaining in the papers. The treatment of filter paper with TFT strictly followed procedures of a previous study that established the initial concentrations [40]. Assuming the rate of volatility is constant regardless of concentration applied and the remaining residual concentrations at time of testing elicited 'sublethal' effects, the ER actions were comparable as a ‘relative’ dose-response between the four different concentrations. From the data, it was apparent there was sufficient AI remaining on the papers at 24 hours to produce marked escape responses.

The relatively high vapor pressure chemistry of TFT compared to other pyrethroids, e.g., deltamethrin and permethrin, may require the use of treated papers in tests much closer to time for treatment. Alternatively, if wanting to link actual dose with response, when repeating similar experimentation, the preferred method is to analyze the amount of AI present in the papers at the time of testing. The optimum time-lag between treatment and testing procedures needs to be explored further and verified for comparing test standardization and interpretation of findings with other compounds with high vapor pressure properties (e.g., metofluthrin). Additionally, the physical/chemical effects of silicon oil (diluent) on the rate of vaporization needs clarification. Until there are a determination and agreement on preferred testing procedures, we provide a cautionary note when using treated papers in testing systems for the evaluation of TFT and similar compounds against insects.

The degree of contact irritability exhibited by mosquitoes can vary according to species and the type and dose of insecticide used [27, 25]. Higher concentrations of pyrethroids can increase the contact escape response of *Ae*. *aegypti* [32] and *Culex quinquefasciatus* Say [29]. Contrastingly, in this study, a decline in escape was observed in *An*. *minimus* with increasing concentration when allowed direct contact with TFT, due to the stronger knockdown effect inside the chamber (non-escaping mosquitoes). A notable decrease in escape was also observed in *Ae*. *aegypti* between the three lower and highest concentrations, while *An*. *dirus* had the highest percent escape at the lowest concentration. The reduced escape is attributed to the stronger knockdown effect inside the chamber (non-escaping mosquitoes). In noncontact tests, colonized *Ae*. *aegypti* escape was greater at concentrations above the LC_50_. Although the repellency escape in DDC and TMMU anopheline strains tended to decline with increased concentration, all but one pairing (LC_75_ vs. LC_99_) found differences were not significant. Overall, both *Anopheles* species showed significantly stronger escape responses compared to *Ae*. *aegypti* (statistics not shown). Nentwig et al. [46] reported positive correlation with increased repellency and concentration of TFT in *Ae*. *aegypti*. Estrada et al. [25] also reported similar repellency response (20–40%) for both *Ae*. *aegypti* and *Ae*. *albopictus* with increasing TFT concentrations ranging from 0.0005% to 10% using the high-throughput screening system.

The ER contact test design potentially measures the combined contact and noncontact mechansims of behavioral avoidance. Unlike the noncontact test design that measures spatial repellency only, the contact test cannot definitively separate between excitation and repellency responses. To estimate the effect of physical contact with a treated surface, mean contact escape responses were adjusted a second time (see Materials & Methods) using the concurrent paired noncontact escape measurements. The resulting adjustment showed TFT produced strong spatial repellency in colonized *An*. *minimus* and *An*. *dirus*. Contact excitation played a much larger role with colonized *Ae*. *aegypti* (USDA) with LC_50_, more comparable with repellency produced by LC_75_ and LC_99_, and a reduced excitation response for the DC. Conversely, using the LC_50_, the *Ae*. *aegypti* (NON) field population showed no contact response. Nentwig et al. [46] also showed TFT exhibited consistently high spatial repellency against *Ae*. *aegypti* using three difference tests systems (Y-olfactometer: >80%, double cage system: 60–80%, and double room system: >80%). Wagman et al. [47] also reported that sublethal doses of TFT produced spatial repellency behaviors in mosquitoes by airborne-induced neurotoxic ‘irritation’ (stimulation).

The adjusted contact data showed colonized and field population anophelines produced relatively poor contact excitation compared to marked repellent activity. The robust repellency response seen with TFT is in contrast to other less volatile pyrethroids. Using the same ER assay system and a range of different pyrethroids, including deltamethrin, bifenthrin, permethrin, α-cypermethrin and λ-cyhalothrin, contact excitation appears the predominant mode of action for *Aedes*, *Anopheles* and *Culex* mosquitoes [28, 29, 31, 35, 36]. Chemically, TFT is more volatile than most conventional pyrethroids and readily vaporizes at room temperature which may explain the variations in action [21].

Beside ER responses, mosquitoes encountering TFT can be knocked down (KD) or can be killed, depending on the exposure and species [46, 48]. Martin et al. [48] showed that *An*. *minimus* was more susceptible to KD and mortality than *An*. *dirus* when exposed to 2% TFT for 60-min in a large semi-field enclosure (28 m long, 3 m wide and 3 m high). However, in this study, *An*. *dirus* showed much greater KD and mortality compared to *An*. *minimus*, while *Ae*. *aegypti* was less sensitive to KD (except for the DC). Exposing to TFT for 30-min or less (mosquito escaped from the treated chamber) may result in variable KD response for each mosquito species. Additionally, mosquitoes recovered within 24-h with decreased mortality in all treatment (except for *Ae*. *agypti* at LC_50_). In this study, spatial repellency of TFT occurred more quickly than a toxic response at test concentrations by causing mosquitoes to escape from the chamber. However, as shown in the recovery of the non-escape groups from knockdown, the test concentrations may not be high enough to completely kill all mosquitoes. As exposure time is one of the important criteria for evaluating the chemical action [26], further study could investigate the effects of varying exposure time on mosquito responses for different TFT concentration.

Regardless of concentration tested, there was no significant difference in escape responses in *An*. *minimus* in the noncontact tests. This finding suggests that lower sublethal concentrations (e.g., LC_50_) TFT may be sufficient in producing strong spatial protection with low knockdown in ceratin mosquitoes (e.g., *Anopheles*). Grieco et al. [26] suggested that the toxic effect of a chemical (e.g. dieldrin) can have a dramatic effect by reducing the population density, but it also carries with it the chance for rapid build up of resistance. Thus, applying the minimal concentration to elicit only a desired behavioral response (avoidance), instead of killing through toxicity at high concentration, might mitigate the development of resistance in insect populations. However, the concentration of volatile chemical required to produce high levels of repellency depends, in part, on the characteristics of the test system used [46]. Mongkalangoon et al. [32] also documented that the LC_50_ of deltamethrin, cyphenothrin, d-tetramethrin and tetramethrin were optimal concentrations for repelling *Ae*. *aegypti*. Therefore, this was the rationale for using the single TFT LC_50_ for field populations of *Ae*. *aegypti* and *An*. *harrisoni* in the ER assay system.

At time of testing, the field-collected *Ae*. *aegypti* from Nonthaburi Province was resistant to TFT, while *An*. *harrisoni* from Kanchanaburi Province was fully susceptible at the discriminating concentration [40]. Significantly stronger escape behavior was observed in the *Ae*. *aegypti* NON population compared to the susceptible USDA strain. The USDA strain had a very low repellency response at LC_50_ compared to contact excitation, while the opposite was observed for the NON population. Similarly, Kongmee et al. [31] found that deltamethrin-resistant *Ae*. *aegypti* escaped the deltamethrin-treated ER chamber faster than a susceptible population. Conversely, Boonyuan et al. [29] reported a greater number of colonized permethrin-susceptible *Cx*. *quinquefasciatus* escaped compared to four resistant field populations. Thanispong et al. [49] observed *Ae*. *aegypti* from various locations in Thailand had similar behavioral responses regardless of degree of background resistance to DDT and α-cypermethrin. It has also been suggested that if the chemical (i.e. TFT) can elicit the behavioral responses through spatial repellency and contact excitation actions, then the degree of resistance in certain mosquitoes to a toxic action would be minimal to no effect in disrupting human-mosquito contact [26].

Although the long-standing *An*. *minimus* DDC laboratory strain and recent field *An*. *harrisoni* KAN population, genetically closely related ‘sibling’ species, are susceptible to TFT, a significantly (*P* = 0.0048 and *P* < 0.001 for noncontact and contact, respectively) greater escape response was observed among DDC than KAN mosquitoes. Similarly, DDC had more rapid escape response to bifenthrin (pyrethroid) than recently field-collected *An*. *minimus* [36]. Potikasikorn et al. [33] using *An*. *harrisoni* from same location (Pu Teuy Village) found weak behavioral responses to pyrethroids compared to *An*. *minimus* collected further north in Mae Sot District (Tak Province). Conversely, long-established laboratory strains of *An*. *albimanus* (USDA, Gainesville, FL) [27] and *An*. *dirus* (Armed Forces Research Institute of Medical Science, Bangkok) [28] showed relatively poor behavioral escape action to insecticides compared to more recently colonized and direct field populations of the same species. Under natural settings, wild-caught mosquitoes are much more heterogeneous in age and nutritional/physiological status compared to colonized mosquitoes under controlled conditions. Besides physiological and nutritional conditions that can influence avoidance behavior [50], given other potential factors (e.g., genetical), the interpretation of avoidance responses between inbred strains and more recent field populations should be viewed with caution compared with more homogeneous laboratory strains.

One possible outcome of using a spatial repellent is the diversion of repelled mosquitoes to other areas, thereby possibly increasing transmission risk elsewhere [20, 51]. We acknowledge that TFT as a spatial repellent did not kill mosquitoes and it is unclear whether use of insecticide (e.g., TFT) with repellent effect in households or even larger areas would potentially result in mosquitoes flying to unprotected areas. Maia et al. [51] conducted a 24-week crossover study in Kilombero, Tanzania, comparing the entomological parameters between three villages using 0.03% TFT coils burned outdoors, including a complete coverage village, a partially covered village, and an unprotected village using blank coils only. The authors reported households without TFT coils in the partial coverage village had a significantly higher human blood index for *An*. *arabiensis* compared to houses in the unprotected village. They concluded diversion of *An*. *arabiensis* from repellent users to non-users. Therefore, it is important to be aware of possible diversion when implementing a spatial repellent as a transmission control measure. However, if applying a spatial repellent that able to protect multiple persons over wide areas (e.g., complete coverage area), this could minimize the risk of possible diversionary effects [20].

Understanding the behavioral responses of mosquitoes to insecticides is relevant for supporting current mosquito control tools and development of novel ones. We encourage those investigating chemical properties for the control of mosquito-borne pathogens to include detailed observations on behavioral responses as well as toxicity. The use of varying concentrations of insecticide is preferred to observe the sublethal avoidance responses to each, especially so in spatial repellancy assays. In our view, even in the presence of significant physiological resistance, if an insecticide remains effective for controlling disease transmission by preventing human bite exposure via altered behavior it should continue to be part of the abatement arsenal. The ability to evoke strong mosquito avoidance behavior for reducing human-vector contact is invaluable for understanding a chemical’s properties to control transmission.

This study assessed adult female mosquito behavioral responses with exposure to TFT using an ER assay system. These findings demonstrate clear ER responses but also differences in avoidance behavior between mosquito species and conditions (colony *vs*. recent field collection) to TFT. Further studies on mosquitoes from different locations can help optimize currently available chemical-based intervention tools and spur the development of spatial repellency products using TFT and similar vapor-active compounds. Furthermore, both indoor and outdoor evaluations of TFT action protecting against mosquito bites using different delivery formats is required under natural settings.

## Supporting information

S1 Table Pairwise log-rank comparisons of escape responses between treatment for each concentration of transfluthrin. (*) significant difference at 95% CI; LC, lethal concentration; DC, discriminating concentration; NC; noncontact control, NT; noncontact treatment, CC; contact control, CT; contact treatment.
(DOCX)

S2 Table Pairwise log-rank comparisons of escape responses between noncontact and contact of control group for each concentration of transfluthrin. LC, lethal concentration; DC, discriminating concentration.
(DOCX)
