# Supplementary material for: Behavioral responses to transfluthrin by Aedes aegypti, Anopheles minimus, Anopheles harrisoni, and Anopheles dirus (Diptera: Culicidae)
Source: PLoS One. 2020 Aug 12;15(8):e0237353. doi: 10.1371/journal.pone.0237353 (PMC7423148; doi:10.1371/journal.pone.0237353)
Supplement: S1 Table — (*) significant difference at 95% CI; LC, lethal concentration; DC, discriminating concentration; NC; noncontact control, NT; noncontact treatment, CC; contact control, CT; contact treatment. (DOCX) [file pone.0237353.s001.docx]

S1 Table. Pairwise log-rank comparisons of escape responses between **treatment** for each concentration of **transfluthrin**.

| Mosquito species | Conc. | *P*-value | | |
| --- | --- | --- | --- | --- |
|  |  | NC vs NT | CC vs CT | NT vs CT |
| *Ae. aegypti* | LC_50_ | 0.0807 | <0.0001* | <0.0001* |
| (USDA) | LC_75_ | 0.0002* | <0.0001* | 0.0030* |
|  | LC_99_ | 0.0014* | <0.0001* | <0.0001* |
|  | DC | <0.0001* | <0.0001* | 0.4596 |
| *An. minimus* | LC_50_ | <0.0001* | <0.0001* | 0.1225 |
| (DDC) | LC_75_ | <0.0001* | <0.0001* | 0.0849 |
|  | LC_99_ | <0.0001* | <0.0001* | 0.9303 |
|  | DC | <0.0001* | <0.0001* | 0.0906 |
| *An. dirus* | LC_50_ | <0.0001* | <0.0001* | 0.6561 |
| (TMMU) | LC_75_ | <0.0001* | <0.0001* | 0.8953 |
|  | LC_99_ | <0.0001* | <0.0001* | 0.0038* |
|  | DC | <0.0001* | <0.0001* | 0.4742 |

(*) significant difference at 95% CI; LC, lethal concentration; DC, discriminating concentration; NC; noncontact control, NT; noncontact treatment, CC; contact control, CT; contact treatment.
