# Supplementary material for: Behavioral responses to transfluthrin by Aedes aegypti, Anopheles minimus, Anopheles harrisoni, and Anopheles dirus (Diptera: Culicidae)
Source: PLoS One. 2020 Aug 12;15(8):e0237353. doi: 10.1371/journal.pone.0237353 (PMC7423148; doi:10.1371/journal.pone.0237353)
Supplement: S2 Table — LC, lethal concentration; DC, discriminating concentration. (DOCX) [file pone.0237353.s002.docx]

**S2 Table. Pairwise log-rank comparisons of escape responses between noncontact and contact of control group for each concentration of transfluthrin.**

| **Conc.** | ***Ae aegypti*** | ***An. minimus*** | ***An. dirus*** | ***Ae. aegypti*** | ***An. harrisoni*** |
| --- | --- | --- | --- | --- | --- |
|  | **USDA** | **DDC** | **TMMU** | **NON** | **KAN** |
| LC_50_ | 1.0000 | 0.1354 | 0.7961 | 0.5703 | 0.1196 |
| LC_75_ | 0.1680 | 0.7853 | 0.5535 |  |  |
| LC_99_ | 0.7153 | 0.4733 | 0.4207 |  |  |
| DC | 0.7098 | 0.5131 | 0.3615 |  |  |

LC, lethal concentration; DC, discriminating concentration.
